# Real-World Application of Digital Morphology Analyzers: Practical Issues and Challenges in Clinical Laboratories

**DOI:** 10.3390/diagnostics15060677

**Published:** 2025-03-10

**Authors:** Hanah Kim, Mina Hur, Giuseppe d’Onofrio, Gina Zini

**Affiliations:** 1 Department of Laboratory Medicine, Konkuk University School of Medicine, Seoul 05030, Republic of Korea; md.hkim@gmail.com; 2 Department of Hematology, Università Cattolica del S. Cuore, 00168 Rome, Italy; giusdono@gmail.com; 3 Department of Hematology, Università Cattolica del S. Cuore-Fondazione Policlinico Gemelli, 00168 Rome, Italy; ginazini@gmail.com

**Keywords:** digital morphology analyzers, peripheral blood smears, bone marrow aspirates, body fluid, clinical laboratory, practical issues

## Abstract

Digital morphology (DM) analyzers have advanced clinical hematology laboratories by enhancing the efficiency and precision of peripheral blood (PB) smear analysis. This review explores the real-world application of DM analyzers with their benefits and challenges by focusing on PB smear analysis and less common analyses, such as bone marrow (BM) aspirates and body fluids (BFs). DM analyzers may automate blood cell classification and assessment, reduce manual effort, and provide consistent results. However, recognizing rare and dysplastic cells remains challenging due to variable algorithmic performances, which affect diagnostic reliability. The quality of blood film as well as staining techniques significantly influence the accuracy of DM analyzers, and poor-quality samples may lead to errors. In spite of reduced inter-observer variability compared with manual counting, an expert’s review is still needed for complex cases with atypical cells. DM analyzers are less effective in BM aspirates and BF examinations because of their higher complexity and inconsistent sample preparation compared with PB smears. This technology relies heavily on artificial intelligence (AI)-based pre-classifications, which require extensive, well-annotated datasets for improved accuracy. The performance variation across platforms in BM aspirates and rare-cell analysis highlights the need for AI algorithm advancements and DM analysis standardization. Future clinical practice integration will likely combine advanced digital platforms with skilled oversight to enhance diagnostic workflow in hematology laboratories. Ongoing research aims to develop robust and validated AI models for broader clinical applications and to overcome the current limitations of DM analyzers. As technology evolves, DM analyzers are set to transform laboratory efficiency and diagnostic precision in hematology.

## 1. Introduction

Automated hematology analyzers (AHAs) have brought about significant advancement in the field of clinical hematology laboratories by providing high-quality analytical performances, expanded capabilities, and a broader range of information for complete blood count (CBC) [1,2,3]. In spite of these technical advances, microscopic examination of peripheral blood (PB) smears is still essential and complementary in achieving comprehensive reporting and validating distributional and/or morphologic abnormalities [1,3,4]. Microscopic examinations of blood cells, including their quantity, morphology, size, and abnormalities on PB smears and bone marrow (BM) aspirates, remains inevitable for diagnosing benign and neoplastic hematologic diseases [1,5,6].

Although microscopic examination is the gold standard, it is labor-intensive, time-consuming, expensive, and highly dependent on experienced personnel [1,4]. Moreover, the microscopic examination may exhibit inter-observer variability [7,8]; storing and accessing glass slides poses challenges regarding the availability of space and PB smear reviews [9]. Given these limitations of microscopic examination and the growing demand for efficient solutions, integrating an automated image analyzer system with a fully AHA platform has emerged as a promising approach in hematology laboratories [10,11]. Early development of digital morphology (DM) analyzers can be traced back to the 1960s with systems such as CELLSCAN, Hematrak, and the Cydac Scanning Microscope System. However, these early systems had limited automation and workflow improvements [4,12]. With remarkable technical advances, DM analyzers have emerged as valuable tools for the automated analysis of PB smears, complementing the traditional microscopic examination [4,13,14,15]. Rapid advancements in digital imaging, artificial intelligence (AI), and machine learning (ML) algorithms have facilitated the development of DM analyzers to pre-classify cells, enabling automated analysis, faster slide reviews, and remote transfer of images to central laboratories for review [4,5,15,16,17,18]. DM analyzers have the potential to standardize and enhance the accuracy of cell analysis, thereby improving the diagnostic efficacy in hematology laboratories [4,6]. However, DM analyzers also have limitations, and microscopic examinations are still necessary. This review describes real-world applications of DM analyzers, focusing on the practical issues and challenges in clinical hematology laboratories. In addition to the implementation of DM analyzers, there is room for further optimization of the laboratory workflow with maximized utilization of DM analyzers.

## 2. Currently Available Platforms and Guidelines

As of October 2023, 20 platforms are commercially available in clinical hematology workflows to assist hematopathologists or medical laboratory technologists in extracting information from PB or BM samples, and the US FDA has cleared 17 AI- and ML-enabled devices [4,18,19]. Excluding equipment discontinued from the market, the general information of commercially available DM analyzers for PB smear analysis is summarized in Table 1. According to a recent survey conducted in France, the most frequently introduced platforms in clinical hematology laboratories are CellaVision DM1200 (CellaVision AB, Lund, Sweden) and Sysmex DI-60 (Sysmex Corp., Kobe, Japan), which is based on the CellaVision DM1200 platform; most DM analyzers have been introduced in large-volume laboratories [20,21]. Consequently, most peer-reviewed studies have been performed using the Cellavision and Sysmex platforms [4,20]. Some DM analyzers, such as DC-1, were developed for targeting small-volume laboratories, and the performance of DC-1 has been explored in previous studies [22,23]. The Cobas m511 integrated hematology analyzer (Roche Diagnostics Operations Inc., Boston, MA, USA), which combined a slide maker, slide stainer, digital image-based cell locator, cell counter, and cell classifier into one system, has been introduced, but it is not available on the market [24,25].

In 2014, the International Council for Standardization in Hematology (ICSH) published guidelines for evaluating blood cell analyzers, including DM analyzers, which updated the previous guidelines that were published in 1994 [26,27]. The ICSH also published a review and recommendation for DM analyzers, focusing on platforms, appropriate use, standardization, practical recommendations, validation, and verification [4,26,27]. DM analyzers have been cleared by the US FDA as a “pre-classifier” for verification by a skilled operator, with the operator performing the final classification of all cells. These DM analyzers rely heavily on the blood film quality and stain quality for accurate cell identification [4]. Manual blood films must have a clear feather edge to find cells, while automated blood films are typically more consistently produced and stained [27,28]. However, it is also important to recognize that some automated slide makers can cause traumatic morphological changes, which may lead to errors, especially when operated by unskilled personnel [28]. DM analyzers are designed to work with dedicated staining protocols, and manufacturers may provide recommendations for settings or instrument protocols to assess the individual laboratory’s current slide-making and staining protocols. These guidelines suggest conducting a thorough assessment of all performance characteristics, encompassing precision, accuracy, reproducibility, and the capacity to handle extreme cases like rouleaux, cold agglutinins, red blood cells (RBCs) agglutination, and morphological anomalies [4,27].

## 3. Analysis of PB Smears

A PB smear is the basic sample type used in DM analyzers. There are three types of microscopic examinations for PB smears: a blood smear scan, a blood smear examination with manual differential of white blood cells (WBCs), and a blood smear review [5]. Currently, most DM analyzers are designed to locate and display images of WBCs, RBCs, and platelets (PLTs) from fixed and stained PB smears and are intended to help qualified laboratory personnel perform WBC differentials, RBC morphology characterizations, and PLT count estimations based on these images [29].

### 3.1. WBC Analysis

A WBC pre-classification of representative DM analyzers for PB smears is summarized in Table 2. A WBC analysis by DM analyzers has some weaknesses that arise from several factors.

(1) Limited AI comprehension for selecting fields and cells:

The ability to accurately select fields and cells for review is crucial when analyzing PB smears [4,5,30]. Skilled technologists can choose the most suitable fields for WBC differentials, ensuring a comprehensive assessment of blood cells [31]. However, DM analyzers often automate this process using predefined algorithms and AI, which may not select abnormal cells that are located at the edges or feathered ends [30,32]. Although experienced technologists have the flexibility to adjust their focus to specific areas of interest, DM analyzers lack such a manual microscopy’s flexibility. This may result in inaccurate replication of manual reviews in challenging or ambiguous cases, and the trajectory would be remarkably different, with the risk of false negative findings. In 2020, the full-field imaging of the monolayer and the feathered edge at 100× magnification became available on the X100HT with Slide Loader with Full-Field PBS Application (X100HT, Scopio Labs, Tel Aviv, Israel), being both US FDA-cleared and CE-marked [33]. The US FDA-cleared X100HT locates and displays blood cell images from stained smears, aiding technologists in WBC differential, RBC morphology, and PLT estimation analyses.

(2) Quality of the blood film and stain:

The performance of DM analyzers in identifying and classifying cells can be largely affected by the quality of the blood film and stain. Properly prepared PB smears are essential for assessing cellular morphology and maximizing the diagnostic information from the slide review [28]. Poor-quality blood films and stains can interfere with the DM analyzers’ performance to accurately analyze cells [34]. Several automated slide makers and strainers have been introduced in clinical laboratories, and they prepare and stain PB smears according to the laboratories’ own established criteria [28]. Automated blood films are generally more consistently prepared and stained than manually prepared blood films [27]. One study showed that automatic wedge smear preparation may cause traumatic morphological changes in blood cells, and these artifacts can puzzle morphological evaluation in both traditional and digitized microscopy, by which unskilled operators can be misled [28]. Using different combination sets of slide making and staining, they showed that the automatic procedure is more traumatic for cells than the manual method, and the number of cells with artefacts would be different according to the automatic slide makers. A variable number of artifacts in WBC morphology can be caused by mechanical trauma in automated blood films; traumatic damage to cells, such as the hypo-granularity of neutrophils and pseudopod of lymphocytes, can mimic the morphological manifestations of dysplasia [28]. DM analyzers are designed to work with dedicated staining protocols, and the manufacturers may recommend specific settings or provide an instrument protocol to laboratories. One study explored the applicability of different staining methods to a DM analyzer [35]; the three different staining methods showed a high correlation for neutrophils and lymphocytes but varied for other WBCs. In addition, each staining method varied in terms of detailed analytical performances and potential impact on laboratory efficiency [35]. Based on such findings, each laboratory should further adjust conventional staining methods to extend their applicability to DM analyzers.

(3) Missed or overlooked abnormal cells:

Inaccurate identification of abnormal cells remains still challenging, in spite of advancements in digital imaging and AI algorithms. Specifically, immature granulocytes, eosinophils, and basophils can be difficult to detect accurately using automated methods [36,37]. Inter-observer agreement in identifying normal or hypercellular pathological samples with clear morphology is generally satisfactory. However, disagreements can arise when interpreting dysplastic features, especially when clonal cells are present at low frequencies and below diagnostic threshold levels, resulting in low reproducibility [38,39]. The absence of standardized criteria for classifying dysplasia presents a challenge in developing a universally accurate AI algorithm for recognizing dysplastic cells [15]. Furthermore, similar features in cells of different lineages increase the risk of misidentifying diagnostic cells. DM analyzers may not identify rare or abnormal cells consistently, leading to potential errors in the diagnosis of undetected hematological disorders [12]. It is often challenging to accurately identify and differentiate dysplastic cells with atypical or abnormal morphological features, especially using automated digital imaging and AI algorithms [40]. DM analyzers rely on large datasets of well-annotated cell images to train their AI algorithms, and these algorithms may be biased toward more common cell types. Dysplastic cells are relatively rare in PB smears, making it challenging to obtain a comprehensive dataset with a sufficient number of these cells. As a result, the AI models used in DM analyzers may have limited exposure to dysplastic features, potentially compromising the accuracy of their detection and classification [15]. Furthermore, certain abnormalities, such as smudge cells, can be either artifacts or clinically significant findings, depending on the patients’ clinical context [41].

(4) Reproducibility:

In most papers, the DM analyzers showed satisfactory precision for mature WBCs [22,36,42,43]. However, the precision of DM analyzers for WBC differentials varied considerably across cell classes, even in the same sample [22]. The DM analyzers showed good precision for neutrophils, lymphocytes, and monocytes. In some cases, they still showed poor precision for eosinophils, immature WBCs, and other cells [22,36,43]. Moreover, DM analyzers tended to be more imprecise in leukopenic samples [43].

(5) Leukopenia:

Most DM analyzers are typically set up to count 100 or 200 cells, and the actual counts, including mild leukopenic samples, are usually based on the pre-set value [22]. However, the performance of the DI-60 analysis varied depending on the WBC count; in moderate-to-severe leukopenic samples, fewer cells were counted than the pre-set value [43,44]. In the normal, mild leukopenia, and moderate leukopenia samples, the median cell count per slide was nearly 200 for both manual counting and the DI-60 analysis. However, in the severe leukopenia samples, the cell counts per slide and total cells counted were two-fold and four-fold lower, respectively, when using the DI-60 analysis compared with manual counting (DI-60 vs. manual: 8 cells vs. 16 cells; 91 cells vs. 379 cells) [44]. Additionally, the precision of WBC pre-classification by DI-60 varied for each sample and each cell class, with the percentage coefficient of variation (%CV) being higher for fewer cells per slide [43]. The analysis time (turnaround time, TAT) for pre-classification and verification in DI-60 showed an inverted U shape, increasing in mild leukopenia samples but decreasing in severe leukopenia samples. On the contrary, the TAT for manual counting increased as the number of WBCs decreased, likely because it requires substantially more time to search for cells to count on slides of severe leukopenia samples than on those of samples with a normal WBC count [44]. The short TAT of DI-60 analysis for severe leukopenia samples implies that DI-60 does not actually spend less time on cell counting but counts fewer cells than are counted in the manual method. Moreover, in manual counting, the experts may discriminate between WBCs and non-WBCs, and the latter is not counted. However, DI-60 should acquire images before starting the analysis and pre-classify the cells into WBCs and non-WBCs. Then, non-WBCs, including smudge cells, artifacts, and unidentified cells, should be eliminated during verification. Thus, in severe leukopenia samples, DI-60 may spend substantially more time on cell counting [44]. Therefore, it is recommended that DM analyzers should be evaluated separately for leukopenic samples, even if the overall performance is acceptable. To optimize laboratory efficiency, DM analyzers should be applied selectively depending on the sample type and each laboratory’s situation and only after risk and TAT assessment.

(6) Study protocols of published papers:

The CLSI guidelines (H20-A2) recommend that a minimum of 200 samples be used in studies, with a preference for more cases [45]. Of these, 100 cases should be considered normal (non-diseased), and up to 100 should be selected to meet specific needs. The remaining 100 cases should represent various abnormalities, including infections (acute bacterial, parasitic, or viral), chronic inflammation, aplastic anemia, myeloproliferative neoplasms, or leukemia, with at least 5 to 10 cases of each defined abnormality. Ideally, additional samples should be studied for the wide range of clinical conditions. The samples used in the studies should be selected from the laboratory’s total workload for at least two weeks, including samples from both inpatients and outpatients in various clinical settings. Additionally, the test method should be challenged by the most severe abnormalities that the laboratory may encounter. It may be necessary to actively seek specific abnormal cases and process them using both methods, where they are then randomly inserted as test samples. Although most studies followed the CLSI guidelines, many performance evaluation studies only used normal or a small number of abnormal samples, which may have led to overestimation of the performance of DM analyzers.

### 3.2. RBC Analysis

The RBC morphology characterization of representative DM analyzers is summarized in Table 3. Previous publications on RBC analysis raised the following issues.

(1) Morphology characterization:

The ICSH guidelines recommend a two-tiered grading system for RBC morphologies, categorizing them as either 2+ (moderate) or 3+ (many). Schistocytes are an exception, employing distinct grading systems and threshold percentages at 1+ (<1% of RBCs) due to their clinical significance [46]. The sensitivity of the diagnostic module DM96 varied for different RBC abnormalities, with some showing a 100% sensitivity, such as stomatocytes and sickle cells. In contrast, others had lower rates, such as RBC agglutination and burr cells, at 33% and 47%, respectively. The specificity of DM96 ranged from 84% for schistocytes to 99.5% for sickle cells and stomatocytes [47]. When using the predefined cut-offs in the CellaVision Advanced Red Blood Cell Software (ARBCA), DM96 demonstrated a wide range of sensitivity and specificity for different RBC abnormalities [48]. The DI-60 system demonstrated acceptable accuracy for recognizing polychromasia, target cells, and ovalocytes but had low specificity for schistocytes and anisocytosis despite high sensitivity [49,50,51]. Schistocyte detection is particularly challenging; DM analyzers showed excellent sensitivity but poor specificity, with artifacts sometimes being misidentified as schistocytes [48,49,52]. The ICSH 1+ criterion (<1% of RBCs) for schistocytes may be too sensitive for digital systems, with 2+ potentially being more suitable [52]. Given these discrepancies, the ICSH guidelines, originally developed for manual microscopy, may not be directly applied to digital systems [46,52]. New RBC grading criteria tailored for DM analyzers may be necessary to account for differences in artifact detection, resolution, and analysis principles between manual and digital methods [50,51,52].

(2) Reproducibility:

In the precision analysis of RBC morphology characterization using the DI-60 system, borderline samples containing specific RBCs exhibited inconsistencies in positive results among 20 replicates [50]. It was also observed that the CV values for within-run, between-run, and between-observer measurements were lower when using the DM96 than when using manual counting [49]. The low CV (1.7–14.5%) for microscopic or post-reclassification within- and between-run variabilities contrasted with the relatively high CV (58–78%) for inter-observer variability. Despite using practical definitions proposed by the ICSH Working Group, the variability between 10 observers evaluating the same samples was 58% when using ARBCA; however, this variability was lower than that observed with manual counting (CV = 78%). Moreover, it is anticipated that the easy and clear presentation of all counted schistocytes on a computer screen and its utility as a teaching tool will further decrease inter-observer variability in the future [49].

(3) Malaria detection:

High-quality thick and thin films should be prepared and examined in cases of suspected malaria; if thick films are positive, the species should be determined by examination of a thin film. [53]. For quantification of parasites, a minimum of 1000 RBCs should be examined in different areas of the film and should be repeated daily until no parasites (other than gametocytes) remain. If the presence of malaria parasites is uncertain on a thick film, especially with a strong clinical suspicion, an entire thin film should be examined with a ×100 objective lens, starting with the edges and the tail, where parasitized cells may be more frequent. [53]. There are significant challenges to diagnosing malaria, especially in an area where it is not endemic; given the rarity of cases, laboratory technicians may also have limited experience identifying malaria parasites [54].

In the first study that used CellaVision DM96, intracellular parasites in samples with less than 0.1% parasitemia were detected 63% of the time [55]. Higher parasitemia increased the detection rate by CellaVision DM96, and samples with parasitemia of 2.5% or higher were correctly classified [55]. Park et al. [56] reported a case of P. falciparum malaria that produced a false-negative result in DI-60. Although malarial parasites were detected in 6.3% (0.276 × 106/L) by microscopy (×1000) on a thin film, no malarial parasites were detected in the initial run using DI-60. When the slide was tested 10 times using DI-60, the median value of the pre-classified malarial parasites was 0.05% of 2273 RBCs; DI-60 misclassified most malarial parasites as Pappenheimer bodies, Howell–Jolly bodies, and basophilic stippling. In contrast, the median number of parasites verified by a pathologist was 181 (7.85%). This case highlighted the potential of DI-60 to produce false-negative results due to misclassification of malarial parasites, not only in cases with low parasitemia (<1%) but also in cases with higher parasitemia (6.3%) [56]. DM analyzers may have the potential to detect malaria parasites in routine screening. The limitations of DM analyzers, however, should be considered with caution, especially in cases with low parasitemia as well as in non-endemic settings [57,58,59].

(4) Hemoglobinopathies:

Hemoglobinopathies, including alpha thalassemia, beta thalassemia, and sickle cell disease (SCD), present unique challenges due to their diverse RBC morphologies and overlapping features with other hematological disorders [60,61]. Given that DM analyzers are trained primarily on normocytic samples, it is expected that RBC characterization may be limited in the cases with hemoglobinopathies, and such errors can impact clinical decision-making, particularly in non-experienced laboratory personnel [60,61,62].

Although DM analyzers have demonstrated some potential in thalassemia screening, one of the key challenges in thalassemia is marked RBC destruction, leading to the formation of small RBC fragments that fall within the PLT size range [63]. Because many DM analyzers rely on size-based algorithms for PLT count estimation, these fragments are often misclassified as PLTs, resulting in erroneous overestimation of PLT counts [63]. SCD is characterized by the presence of sickle-shaped RBCs, target cells, poikilocytosis, and schistocytes [47,64]. While DM analyzers exhibited high specificity for sickle cells, pre-classification frequently resulted in high false-negative rates, requiring manual correction by experienced hematologists [47,64]. Previous studies underscore the need for improved AI algorithms and validation protocols; expert validation, incorporating RBC-related flags, RBC indices, and reticulocyte counts from AHAs, remains essential for accurate RBC characterization, especially in cases with mixed RBC abnormalities [60,61,62,63,64].

### 3.3. PLT Counting

In conjunction with AHAs, using DM analyzers may allow for visual identification of PLTs and observation of size, distribution, and cytoplasmic particle abnormalities. Previous studies on the application of DM analyzers in PLT counting have exposed several issues that still need to be studied. Further studies are needed to clarify PLT counting issues on various DM analyzers. The PLT count estimation of DM analyzers is summarized in Table 4.

(1) Field selection and PLT count calculation:

When counting PLTs, the selection of fields is an important factor, and most DM analyzers select only a certain number of fields and their location. PLT clumps are mostly located at the lateral or feathered edge of the PB smear [11,31,35,65]. Currently, most DM analyzers count PLTs by counting PLTs manually in a predetermined number of fields and multiplying the number of PLTs counted by a predetermined constant [10,11,52,66,67]. Therefore, the users and the fields selected would influence these semi-automated PLT counts considerably, leading to over- or underestimation of PLT counts. It has been reported that PLT count estimation by DI-60 was overall satisfactory, with a very high correlation with Sysmex XN analyzer; however, the DI-60 system underestimated PLT counts in samples with marked thrombocytosis [35]. Another study by Kim et al. [11] showed that the discrepancy between the predicted and actual number of PLT counts increased as the PLT counts increased. Thus, applying a uniform pre-set estimation factor, regardless of the PLT counts, might be questionable; instead, it would be more reasonable to adjust the pre-set estimation factor based on the PLT counts [68].

The new MC-80 DM analyzer (Mindray Bio-Medical Electronics, Shenzhen, China) adopted two different principles for PLT count estimation: PLT/RBC ratio (PLT-M1) and estimate factor (PLT-M2). Although these two methods correlated well with the reference method, they may provide inconsistent and biased results, especially related to the RBC count and RBC size [69]. As the RBC count increased, PLT-M1 tended to produce a positive deviation relative to the reference method, while PLT-M2 had a negative deviation. The cause of this bias may be related to the scanning area: when the RBC count in the sample is high, the MC-80 tends to scan towards the tail end of the smear. Consequently, when the RBC count in the blood is high, the RBC and PLT counts on the smear may be somewhat underestimated, leading to a higher PLT-M1 (due to a greater underestimation of RBCs) and a lower PLT-M2. This issue may be improved by expanding the area examined. When small RBCs were present, the two PLT counting results were relatively high, and the correlation with the reference method decreased [69]. Samples with small RBC interference mainly originate from patients with microcytic anemia, where RBCs are easily seen to be overlapping each other at the body–tail junction of the PB smear. Although the MC-80 can automatically target the appropriate region between the body and tail, it may have difficulty identifying the correct region for samples with small RBCs, particularly in anemic samples. This improper targeting may be a factor contributing to the observed bias. The DM analyzers would record two or more RBCs overlapping each other as one RBC.

As mentioned above in the cases with hemoglobinopathies, spurious thrombocytosis can arise from various causes, including fragmented RBCs, microcytic RBCs, fragments of WBCs, bacteria, fungi, lipids, and cryoglobulins. Conversely, spurious thrombocytopenia can also be observed in the presence of large PLTs, exceeding the upper limit of the normal range of PLT size. To confirm these findings, experts should integrate RBC-related flags, RBC indices, and reticulocyte counts from AHAs and assess lipemic interference during validation.

(2) PLT clumps:

In cases where EDTA-induced pseudothrombocytopenia is suspected in blood samples, manual confirmation on a PB smear is imperative, and supplementary procedures or additional blood collection are required to ensure accurate PLT counts. A recent study reported that, in the presence of PLT clumps, the resulting PLT counts determined using the DI-60 analysis were not sufficiently accurate enough to be accepted as the final count [67]. Another study reported that an integration of MC-80 with BC-6800Plus AHA (Mindray Bio-Medical Electronics) enabled accurate PLT counting in samples with EDTA-induced pseudothrombocytopenia [70]. BC-6800Plus has a unique feature of dispersing preformed PLT aggregates, and the sensitivity and specificity of PLT clump flagging by BC-6800Plus significantly increased by incorporating imaging information obtained using the MC-80 [70]. Such a combination was also effective in detecting actual PLT clumps, even in CBCs with blood anticoagulated with sodium citrate. When PLT clumps are present, a final confirmation of the accurate PLT count is still required from an experienced examiner. DM analyzers would be a useful adjunct tool that can provide visual insight into each case.

## 4. Analysis of BM Aspirates

BM examination is the current gold standard for investigating many hematological diseases as well as various non-hematological conditions [71,72,73,74]. DM analyzers for BM aspirate assessment have yet to be widely adopted in clinical settings. The DI-60 and DM1200 systems, which have been used in many laboratories for PB smears, are currently not applicable to BM aspirate specimens. Based on recent advancements in AI-based models, including convolutional neural networks and attention-based algorithms, DM analyzers have emerged as powerful tools offering improved efficiency, accuracy, and standardization in BM aspirate analysis [12,72,75]. AI-based BM evaluation can be integrated into the hematopathology workflow, playing an assistive role and/or triaging role; at the institutional level, it also has the potential to further change the diagnostic system towards a centralized approach (digital send out test) vs. decentralized approach (digital laboratory-developed test) [72].

However, variability in staining techniques and the lack of standardized grading criteria for DM analyzers also highlight the need for further development and validation [66,70]. Two methods are available for analyzing nucleated cells on BM aspirates using DM analyzers. The first method includes hardware and AI-based software like Scopio Labs’s X100 and X100HT with Full-Field Bone Marrow Aspirate™ (FF-BMA) Application (Scopio), Morphogo (Zhiwei Information Technology Co., Ltd., Hangzhou, China), the Metafer Slide Scanning System (MetaSystems Hard & Software GmbH, Altlussheim, Germany), and Vision Bone Marrow (West Medica, Wiener Neudorf, Austria). These products are currently available on the market, and this review will focus on them. The second method utilizes hardware such as TissueScope (Huron Digital Pathology, St. Jacobs, ON, Canada), the Leica Aperio slide scanner (Leica Biosystems, Wetzlar, Germany), or smartphones to capture slide images, including whole slide images, using separated and/or dedicated AI-based software [76,77,78,79,80,81,82,83].

DM analyzers may provide advantages in clinical hematology laboratories, such as reducing workload and remote capability; however, there are still several practical issues. The key specifications of Scopio and Morphogo systems are compared in Table 5.

**Figure 1:**
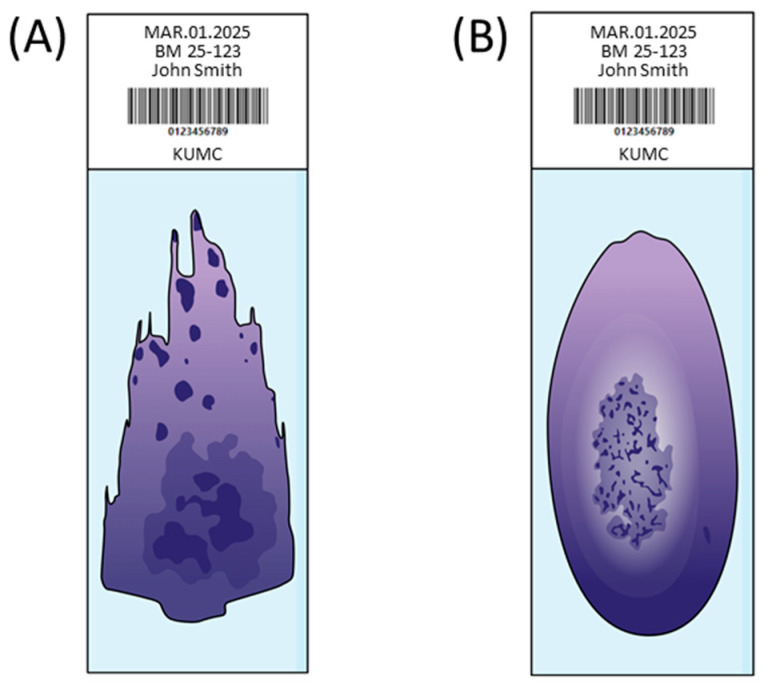
Illustration of bone marrow (BM) aspirate smears. The label contains fabricated information as an example. (**A**) Wedge-spread smear. A small drop of BM particles is placed 1 cm from the slide’s edge. A spreader at a 30-degree angle is used to drag the aspirate forward, creating a smear of 3–5 cm in length. (**B**) Crush technique smear. BM particles are placed in the center of the slide. A second slide, perpendicular to the first, is gently pressed and pulled apart to create the smear.

(1) Variability in smear preparation:

Unlike PB smears, which have a standardized, automated process for creating and staining smears, methods for the preparation, processing, and reporting of BM aspirate smears can vary considerably. These differences may result in inconsistencies in the diagnosis or classification of disease that may affect treatment and clinical outcomes [73]. In addition, preparing for BM aspirate smears require operators’ skill and experience to ensure optimal quality for analysis. Various factors such as smear thickness, staining quality, and artifacts can affect the system’s ability to identify and classify cells accurately [71]. The quality of BM aspirates may impact the performance of DM analyzers significantly. According to the ICSH recommendation, BM aspirate smears should be prepared simultaneously using two distinct techniques: the wedge-spread technique and the crush technique [72,73] (Figure 1). It has been reported that the composition of BM cells may differ significantly between the two techniques, and the crush technique would be more valuable than the wedge-spread technique because of the lack of a blood dilution effect and better assessment of megakaryopoiesis [74].

Staining quality is another critical factor; inconsistent or suboptimal staining can lead to misclassification of cells by both human experts and digital systems. In a recent multicenter study on the Scopio Labs X100 FF-BMA system, the overall agreement between the test and reference method for the BM aspirate assessment was reported to be high; however, the results showed different performance metrics between Romanowsky and Prussian blue-stained BM aspirate smears (89.12% [range, 88.50–89.76%] vs. 83.48% [range, 80.20–86.59%]), with variations across participating laboratories [84].

(2) Classification accuracy for rare cells:

Previous studies have reported that DM analyzers performed well in identifying common cell types on BM aspirate smears. The Metafer4 VSlide system showed excellent agreement for the myeloid and erythroid series, with a mean difference of 0.0 for neutrophil and eosinophil granulocytes, −0.2 for the sum of all myeloid cells, and 1.2 ± 3.0 for the erythropoietic series compared with optical microscope [82]. The Scopio Labs X100 FF-BMA system demonstrated a high correlation with the reference method, with an acceptable accuracy for grading primary maturation and morphology assessment (90.85% efficiency, 81.61% sensitivity, and 92.88% specificity for Romanowsky-stained samples) [84]. The Morphogo system achieved a classification accuracy above 85.7% in a study of 230 cases, with averages of sensitivity and specificity at 69.4% and 97.2%, respectively [85,86]. However, identifying rare myeloid precursors is challenging due to their low frequency and potentially subtle morphological differences from other cell types. Lewis et al. [12] developed an automated pipeline for differential cell counts on whole-slide BM aspirates, which included rare myeloid precursors. However, they noted that the accuracy for rare cell types was lower than that for more common cell types, highlighting the ongoing challenge in this area. Due to their large size and complex morphology, megakaryocytes can be also challenging for DM analyzers. The Morphogo system showed high performance in identifying megakaryocytes in BM aspirate smears (sensitivity, 96.57%; specificity, 89.71%) with a good correlation (r ≥ 0.72) with microscopic examination in classifying megakaryocytes [77]. Regarding dysplastic cell detection, data on the performance of the novel DM analyzer systems are still lacking [79,82]. Therefore, further studies are needed to evaluate the performance of various DM analyzers focused on rare cells and/or dysplastic cells.

(3) Limited standardization:

The complexity of cell types on BM aspirate smears poses significant challenges for the external quality control (QC) of DM analyzers. Unlike PB, BM aspirate samples contain a wide variety of cell types at different stages of maturation and rare and abnormal cells crucial for diagnosing hematological disorders [73]. This diversity makes it difficult to establish standardized reference samples for external QC programs, often leading to inter-platform variability, as different DM analyzers can demonstrate unique performance characteristics [12]. Considering the absence of consensus guidelines or recommendations for good laboratory practice implementing DM analyzers for BM aspirate smear analysis, a lot of effort should focus on standardization and harmonization to guarantee its promising role in the near future.

## 5. Analysis of Body Fluids

Few studies have evaluated DM analyzers on BFs, and like BM aspirates, applying DM analyzers in BFs is still in its infancy. Several DM analyzers offer a BF mode, but the performance is not as satisfactory as a PB smear analysis [87,88,89,90]. Key specifications of several DM analyzers for BF analysis are compared in Table 6. The BF analysis of DI-60 is designed to utilize cytocentrifuge-prepared slides and digitally scan the entire smear area (at 10× or at 50× magnification). The DI-60 analysis pre-classifies a total of eight classes (six WBC classes and two non-WBC classes) in an automatically selected area. In an overview application, examiners can select the area of interest manually and detect abnormal cells more easily [88]. DM analyzers like Sysmex DI-60 may show high overall sensitivity, specificity, and accuracy for common cell types, although its performance can vary depending on the predominant cell types [89,90]. The performance may be less reliable in samples with predominant abnormal lymphocytes or malignant cells, which are classified as ‘other’ cells in DI-60 [90]. Using BF samples with a single, dominant cell type, Shin et al. [90] also reported that the median TAT for DI-60 was significantly longer than manual counting across all sample types, and the TAT varied depending on the predominant cell type in the samples. They inferred that the data in the real-world practice would show more remarkable differences and larger gaps, using various BF samples with heterogenous cell compositions. The prolonged TAT of the DI-60 compared with that of manual counting may leave a critical question about whether current DM analyzers would indeed streamline laboratories’ workflows and improve laboratory efficiency [90]. DM analyzers may be suitable for screening purposes, especially in understaffed laboratories; however, the data in the literature show that the current DM analyzers may not fully replace manual review for all sample types [89]. Further improvement and data accumulation would be necessary before applying DM analyzers for routine clinical practice in BF analysis.

## 6. Related Issues

Currently, there is no standardized external accuracy management using the College of American Pathologists (CAP) or nation-based external quality assessment schemes (EQASs) for the evaluation of DM analyzers. This lack of standardization is a significant challenge in the field of DM analysis [4]. The absence of such QC measures is thought to be due to various technical differences across platforms, such as the standardization of slide production, staining methods, optical magnifications, color and display characteristics, hardware, software, and file formats used for capturing and analyzing images [4]. This lack of EQA significantly challenges ensuring consistent and reliable results across different laboratories. Therefore, the ICSH has highlighted the need for standardization across different DM analyzers to improve the comparability and reliability of results [4]. Grimon, et al. [20] conducted a survey in France that assessed the use of DM analyzers in hematology. Many laboratories did not perform regular QCs, highlighting an urgent need for standardized guidelines. Rosetti, et al. [91] explored the feasibility of incorporating DM analyzers, specifically Sysmex DI-60, into the UKNEQAS EQA program for manual differentials. That study highlighted that the absence of specific EQA samples for DM analyzers limits a thorough validation in real-world scenarios, making it essential to develop dedicated EQA protocols for DM [8,91]. This is not a matter that is just confined to the PB smear analysis but a matter that should be extended to the BF and BM aspirate analysis.

The DM analyzers have shown varied impacts on TAT and workflow efficiency in hematology laboratories [44,92]. A study on remote review using Scopio Labs’s X100 demonstrated substantial TAT reductions, eliminating backlogs and allowing for the removal of an entire shift [93]. When applying the remote review of PBS slides using Scopio Labs’s X100 with Full-Field PBS Application, the overall morphology TAT in the laboratory dropped by 15.8% over a five-month period compared with the previous DM workflow. However, it should be remembered that that analysis was just focused on the remote review, with the TAT analysis being confined to the samples over weekends and the first two weekdays [93]. Moreover, the impact may vary depending on the specific analyzers, the type of samples being processed, and the laboratory setup. The pre-classification process in Sysmex DC-1 demonstrated a faster overall TAT than manual counting [22]; when considering the total TAT and TAT per cell, however, no significant differences were observed between Sysmex DC-1 and the manual counting method [22]. The DI-60 system required more time per cell than manual methods across all leukopenia levels [44]. This extended the TAT in DI-60, especially for the samples with mild to moderate leukopenia, and it was attributed to the complexity of cellular composition, including a diverse range of cell types that increase processing time. The implementation of DM analyzers can lead to more efficient workflows, reduced backlogs, and potential cost savings; however, it may require careful consideration of the specific laboratory needs and sample types.

## 7. Conclusions

The practical application of DM analyzers in hematology laboratories has demonstrated significant advancements in laboratory efficiency, consistency, and diagnostic support [4,6]. DM analyzers have shown promise in reducing inter-observer variability and automating smear analysis, particularly for routine PB smear examinations [20,90]. However, the real-world implementation of DM analyzers also highlights several challenges that warrant continued research and optimization. In addition to the PB smear, complex samples such as BM aspirates and BFs require further algorithmic refinement and accumulation of clinical data [82,87]. Key challenges for the real-world application of DM analyzers in clinical laboratories are summarized in Table 7.

The dependency of DM analyzers on high-quality blood films and staining techniques underscores the necessity of standardization across various platforms [4,91]. Additionally, the limitations in identifying rare and dysplastic cells indicate that skilled manual review remains essential for complex cases [20,40]. As DM analyzers evolve, enhancements in AI and machine learning algorithms will be critical for improving the accuracy of cell classification, particularly for atypical or abnormal cells [20,91].

Future integration of DM analyzers in clinical settings will likely involve a hybrid model, combining automated analysis with expert oversight. This approach may optimize a workflow efficiency while maintaining diagnostic accuracy [41]. To fully leverage DM analyzers’ potential, establishing robust EQA protocols and advancing training datasets will be vital in standardizing the performance and addressing current limitations [4,91]. With ongoing advancements, DM analyzers would hold the potential to transform hematology diagnostics by offering reliable, automated support for clinical decision-making, as demonstrated by improvements in TAT and workflow efficiency in various laboratory settings. However, it should be also acknowledged that there is a lack of large-scale clinical validation studies, leaving room for further research to support these theoretical assumptions on the benefit or utilities of DM analyzers. Laboratory preparedness for the AI-empowered dynamic revolution in the era of digitization cannot be overemphasized. Professional guidelines and consensus recommendations are also inevitable for harnessing DM analyzers in clinical laboratories.

## Figures and Tables

**Table 1:** General information of commercially available digital morphology analyzers for peripheral blood smear.

	DM 1200	DM 9600	DI-60	DC-1	MC-80	Scopio X100 (X100HT)	VISION Hema *
Manufacturer	Cellavision, Lund, Sweden	Cellavision, Lund, Sweden	Cellavision, Lund, Sweden	Cellavision, Lund, Sweden	Mindray, Shenzen, China	Scopio Labs, Tel Aviv-Yafo, Israel	West Medica, Wiener Neudorf, Austria
Launched year	July 2009	April 2014	2013	February 2019	September 2021	April 2020 (May 2022)	NI
US FDA approval	Yes	Yes	Yes	Yes	No	Yes	No
Intended use	WBC differential, characterization of RBC morphology and PLT estimation	WBC differential, presenting overview image of RBC and PLT	WBC differential, presenting overview image of RBC and PLT	WBC differential, characterization of RBC morphology, and PLT estimation	WBC pre-classification, pre-characterization of RBC morphology, and PLT estimation	WBC differential, RBC morphology evaluation, and PLT estimation	WBC identification/pre-classification, RBC analysis, and PLT analysis
CE-marked	Yes	Yes	Yes	Yes	Yes	Yes	Yes
Throughput	20 slides/h for 100 WBC + RBC + PLT	30 slides/h for 100 WBC + RBC + PLT	30 slides/h for 100 WBC + RBC + PLT	10 slides/h for 100 WBC + RBC + PLT	60 slides/h for 100 WBC + RBC + PLT	15 slides/h for 200 WBC differential (40 slides/h)	NI
Supported stains **	Romanowsky, RAL, MCDh	Romanowsky, RAL, MCDh	Romanowsky, RAL, MCDh	Romanowsky, RAL, MCDh	Romanowsky	Romanowsky	Romanowsky
Installation type	Stand-alone	Stand-alone	Integrated with Sysmex XN-series	Stand-alone or networked installation	Integrated with Mindray’s CAL 6000 and CAL 8000	Stand-alone	Stand-alone

* VISION Hema is a clinical application module that is applied to the three analyzers (Vision Assist, Vision Pro, and Vision Ultimate). ** Romanowsky stains include May-Grünwald Giemsa, Wright-Giemsa, and Wright stains. Abbreviations: FDA, Food and Drug Administration; h, hour; MCDh, micro chromatic detection for hematology; NI, no information; PLT, platelet; RBC, red blood cell; WBC, white blood cell.

**Table 2:** WBC pre-classification of digital morphology analyzers for peripheral blood smears.

	DM 1200	DM 9600	DI-60	DC-1	MC-80	Scopio X100 (X100HT)	VISION Hema
Number of FOVs	Acquisition to obtain 3 times the requested WBC count	Acquisition to obtain 3 times the requested WBC count	Acquisition to obtain 3 times the requested WBC count	Acquisition to obtain 3 times the requested WBC count	HPF instead of FOVs	WSI	NI
Total class	18	18	18	18	27	24	16
WBC class	13	13	13	13	21	14	13
Pre-classification	Seg. N, band N, L, M, E, B, metamyelocytes, myelocytes, promyelocytes, blasts, plasma cells, variant form, unidentified *	Seg. N, band N, L, M, E, B, metamyelocytes, myelocytes, promyelocytes, blasts, plasma cells, variant form, unidentified *	Seg. N, band N, L, M, E, B, metamyelocytes, myelocytes, promyelocytes, blasts, plasma cells, variant form, unidentified *	Seg. N, band N, L, M, E, B metamyelocytes, myelocytes, promyelocytes, blasts, plasma cells, variant form, unidentified *	Seg. N, band N, L, M, E, B, metamyelocytes, myelocytes, prolymphocytes, promyelocytes, promonocytes, blasts, abnormal L, abnormal promyelocytes, centrocytes, hairy cells, large granular L, plasma cells, reactive L, Sezary cells, splenic villous L	Seg. N, band N, L, M, E, B, metamyelocytes, myelocytes, promyelocytes, blasts, aberrant L, atypical L, large granular L, plasma cells	Seg. N, band N, L, M, E, B, metamyelocytes, myelocytes, promyelocytes, blasts, erythroblasts, reactive L, others
Non-WBC class	5	5	5	5	6	10	3
Pre-classification	Artifacts, giant PLT, NRBC, PLT agg, smudge	Artifacts, giant PLT, NRBC, PLT agg, smudge	Artifacts, giant PLT, NRBC, PLT agg, smudge	Artifacts, giant PLT, NRBC, PLT agg, smudge	Erythroblasts, giant PLT, large clumps, large PLT, NRBC, PLT clumps	Artifacts, hairy cell, immature B, immature E, NRBC, prolymphocyte, promonocyte, Sezary cell, smudge, others	PLT agg, smudge, others

* The term “unidentified” is now classified separately from both WBCs and non-WBCs. Abbreviations: FOV, field of view; HPF, high-power field; NI, no information; N, neutrophils; L, lymphocytes; M, monocytes; E, eosinophils; B, basophils; NRBC, nucleated red blood cell; PLT, platelet or thrombocyte; agg, aggregation; Seg. Segmented; WBC, white blood cell; WSI, whole slide image.

**Table 3:** RBC morphology characterization of digital morphology analyzers for peripheral blood smear.

	DM 1200	DM 9600	DI-60	DC-1	MC-80	Scopio X100 (X100HT)	VISION Hema
Number of FOVs	35	35	35	NI	HPF instead of FOVs	1000	NI
Size class	3	3	3	3	3	3	3
Pre-characterization	Anisocytosis, microcytosis, macrocytosis	Anisocytosis, microcytosis, macrocytosis	Anisocytosis, microcytosis, macrocytosis	Anisocytosis, microcytosis, macrocytosis	Anisocytosis, microcytosis, macrocytosis	Anisocytosis, microcytosis, macrocytosis	Anisocytosis, microcytosis, macrocytosis
Color class	2	2	2	2	2	2	3
Pre-characterization	Polychromasia, hypochromasia	Polychromasia, hypochromasia	Polychromasia, hypochromasia	Polychromasia, hypochromasia	Polychromasia, hypochromasia	Polychromasia, hypochromasia	Polychromasia, hypochromasia hyperchromasia
Shape class	1 + 11 *	1 + 11 *	1 + 11 *	1	14	14	12
Pre-characterization	Poikilocytosis acanthocytes *, elliptocytes *, echinocytes *, helmet cells *, ovalocytes *, schistocytes *, sickle cells *, spherocytes *, stomatocytes *, target cells *, tear drop cells *	Poikilocytosis acanthocytes *, elliptocytes *, echinocytes *, helmet cells *, ovalocytes *, schistocytes *, sickle cells *, spherocytes *, stomatocytes *, target cells *, tear drop cells *	Poikilocytosis acanthocytes *, elliptocytes *, echinocytes *, helmet cells *, ovalocytes *, schistocytes *, sickle cells *, spherocytes *, stomatocytes *, target cells *, tear drop cells *	Poikilocytosis	Poikilocytosis, acanthocytes, bite cells, blister cells, echinocytes, elliptocytes, irreg. contracted cells, ovalocytes, schistocytes, sickle cells, spherocytes, stomatocytes, target cells, tear drop cells	Poikilocytosis, acanthocytes, bite cells, blister cells, echinocytes, elliptocytes, helmet cells, ovalocytes, schistocytes, sickle cells, spherocytes, stomatocytes, target cells, tear drop cells	Poikilocytosis, acanthocytes, echinocytes, elliptocytes, helmet cells, ovalocytes, schistocytes, sickle cells, spherocytes, stomatocytes, target cells, tear drop cells
Inclusion class	4	4	4	NA	6	3	5
Pre-characterization	Baso. stippling *, H–J bodies *, P-H bodies *, parasites *	Baso. stippling *, H–J bodies *, P-H bodies *, parasites *	Baso. stippling *, H–J bodies *, P-H bodies *, parasites *		Agglutination, Baso. stippling, H–J bodies, P-H bodies, parasites, rouleaux	H–J bodies, micro-organisms, P-H bodies	Baso. stippling, Cabot rings, H–J bodies, P-H bodies, parasites

* Available with the optional advanced RBC application. Abbreviations: Baso., basophilic; FOV, field of view; HPF, high-power field; H–J, Howell–Jolly; irreg., irregular; NA, not available; NI, no information; P-H, Pappenheimer; RBC, red blood cell; rouleaux, rouleaux formation.

**Table 4:** Platelet count estimation of digital morphology analyzers for peripheral blood smear.

	DM 1200	DM 9600	DI-60	DC-1	MC-80	Scopio X100 (X100HT)	VISION Hema
Estimation method	Manual count with pre-set estimation factor	Manual count with pre-set estimation factor	Manual count with pre-set estimation factor	Manual count with pre-set estimation factor	Automatic count with PLT/RBC ratio (recommended), manual count with pre-set estimation factor	Automatic count with pre-set estimation factor	Manual count with pre-set estimation factor
Number of FOVs	35	35	35	NI	HPF instead of FOVs	10	NI
Default estimation factor *	0	0	0	0	4.462 (3 HPF) 2.677 (5 HPF) 1.339 (10 HPF)	10,000	NI
Corresponding HPF	8 HPF (100×)	8 HPF (100×)	8 HPF (100×)	8 HPF (100×)	3, 5, and 10 HPF	10 HPF	NI
Size	NA	NA	NA	NA	NA	NA	Normal, micro, macro
Clumps	Yes ^†^	Yes ^†^	Yes ^†^	Yes ^†^	Yes	Yes	No

* All manufacturers’ default estimation factors are adjustable, and each laboratory should determine the pre-set estimation factor during installation. ^†^ Available on the latest software version (ver.7.1). Abbreviations: FOV, field of view; HPF, high-power field; NA, not available; NI, no information.

**Table 5:** Comparison of key specifications of digital morphology analyzers for bone marrow aspirates.

	Scopio X100 or X100HT	Morphogo
Manufacturer	Scopio Labs, Tel Aviv-Yafo, Israel	ZhiWei Information & Technology Co., Ltd Hangzhou, China
Launched year	September 2022	May 2022
US FDA approval	Yes	No
Intended use	Automatically locates and presents images of hematopoietic cells, quality assessment, blast cell, plasma cell, and M:E ratio estimation	No
CE-marked	Yes	Yes
Technology	Full-field cell morphology AI-powered DSS	Whole slide image CNN-based AI system
Throughput	X100: up to 3 slides/h X100HT: up to 9 slides/h	Up to 8 slides/h (500 nucleated cells + all megakaryocytes)
Magnification	Up to 100× (full field)	Up to 100× (100×: ROI only)
Oiling Method	X100: manual dispensing X100HT: automatic dispensing	Automatic dispensing
Discovering ROI	No need to setup ROI for review, 100× Full-field review	Whole slide review by 40×, ROI review by 100×
Slide preparation technique	Wedge spread, squash, touch print	Wedge spread
Supported stains	Romanowsky, May-Grünwald, Giemsa, Wright, Wright-Giemsa, Prussian blue (for iron store evaluation)	Romanowsky, May-Grünwald, Giemsa, Wright, Wright-Giemsa
Number of nucleated cell pre-classified	300, 500 (default), or 1000	Up to 9999
Scan time	Scopio X100: 20 min Scopio X100HT: 7 min	6 min (40× whole slide imaging with all megakaryocytes detected + 200 nucleated cell pre-classified under 100×)
Specimen quality check	Particle presence (particulate/pauciparticulate/aparticulate), precursor cell presence (present/not present), specimen quality (adequate/hemodiluted/inadequate [dry tap]) (DSS)	Qualitative (cellularity, particle presence)
Blast count estimation	Quantitative (pre-classified)	Quantitative (pre-classified)
Blast morphology	Auer rods (yes [single/multiple]/no), granulation detection (yes/no), nucleus morphology (normal/abnormal) (manual)	NA
M:E ratio	Quantitative (pre-classified)	Quantitative (pre-classified)
Myeloid class	10	14
Pre-classification	Blast, promyeloblast, neutrophilic myelocyte, metamyelocyte, band neutrophil, segmented neutrophil, monocyte, eosinophil, basophil, mast cell	Myeloblast, promyeloblast, neutrophilic myelocyte, neutrophilic metamyelocyte, band neutrophil, segmented neutrophil, eosinophilic myelocyte, eosinophilic metamyelocyte, band eosinophil, segmented eosinophil, basophil, monoblast, promonocyte, monocyte
Erythroid class	4	7
Pre-classification	Erythroblast, basophilic normoblast, polychromatophilic normoblast, normoblast	Proerythroblast, early erythroblast, intermediate erythroblast, late erythroblast, megaloblastic early erythroblast, megaloblastic intermediate erythroblast, megaloblastic late erythroblast
Lymphoid class	1	4
Pre-classification	Lymphocyte	Lymphoblast, prolymphocyte, mature lymphocyte, atypical lymphocyte
Plasma cell class	1	3
Pre-classification	Plasma cell	Plasmablast, immature plasma cell, plasma cell
Others class	2	6
Pre-classification	Unclassified, additional cell (macrophage, others, artifact)	Erythrocyte, histiocyte, mast cell, mitosis, platelet, smudge cell
Megakaryocytes class	1	1
Pre-classification	Megakaryocyte	Megakaryocyte
Prussian blue stain *	Iron store (normal/abnormal), ring sideroblast (normal/abnormal)	NA

* Manual evaluation by users. Abbreviations: DSS, decision support system; h, hour; min, minute; NA, not available; ROI, region of interest.

**Table 6:** Comparison of key specifications of digital morphology analyzers * for body fluid analysis.

	DM 1200	DM 9600	DI-60	MC-80	VISION Hema Body Fluids Application Module
Available sample	CSF, serous fluid, synovial fluid	CSF, serous fluid, synovial fluid	CSF, serous fluid, synovial fluid	CSF, serous fluid, synovial fluid	Exudates, CSF
WBC class	6	6	6	6	11
Pre-classification	Neutrophils, lymphocytes, eosinophils, macrophages (including monocytes), others (basophils, lymphoma cells, atypical lymphocytes, blasts, and tumor cells), unidentified	Neutrophils, lymphocytes, eosinophils, macrophages (including monocytes), others (basophils, lymphoma cells, atypical lymphocytes, blasts, and tumor cells), unidentified	Neutrophils, lymphocytes, eosinophils, macrophages (including monocytes), others (basophils, lymphoma cells, atypical lymphocytes, blasts, and tumor cells), unidentified	Neutrophils, lymphocytes, eosinophils, macrophages (including monocytes), others (basophils, lymphoma cells, atypical lymphocytes, blasts, and tumor cells), unidentified	Neutrophils, lymphocytes, eosinophils, macrophages (including monocytes), plasma cells, atypical cells, mesothelial cells, ependymal cells, arachnoid cells, cricoid cells, others
Non-WBC class	2	2	2	2	3
Pre-classification	Artifacts, smudge	Artifacts, smudge	Artifacts, smudge	Artifacts, smudge	Artifacts, microorganism, unknown
RBC class	NA	NA	NA	NA	2
Pre-classification	NA	NA	NA	NA	Normal erythrocytes, abnormal erythrocytes

* Neither DC-1 nor Scopio X100 (X100HT) can analyze body fluids. Abbreviations: CSF, cerebrospinal fluid; NA, not available; RBC, red blood cell; WBC, white blood cell.

**Table 7:** Key challenges for the real-world application of DM analyzers in clinical laboratories.

Limited research beyond basic WBC pre-classification
There is a need for large-scale studies on WBC classification that encompasses diverse clinical conditions, including rare cells and dysplastic cells. More fundamental research on RBC morphological abnormalities and PLT counting is needed, along with large-scale studies covering a wide range of RBC and PLT abnormalities.
Restricted performance for PB examination
DM analyzers function as pre-classifiers, currently performing at a level comparable with blood smear scans with WBC differentials rather than blood smear examination. The blood smear examination serves three key clinical purposes: (1) verifying automated analyzer results, (2) identifying atypical or immature cells, and (3) detecting critical morphological abnormalities, including RBCs and PLTs that may go unflagged by analyzers [5]. DM analyzers are not yet sufficient enough to fully meet the clinical indications for examination of a blood smear [31].
Early-stage development in BM and BF applications
BM and BF applications of DM analyzers remain in the early stages of research and clinical adoption. The complexity of BM aspirates and BF samples imposes limitations, making them insufficiently reliable for routine use without further advancements in AI-based algorithms and large-scale validation studies.
Need for standardization in EQA and efficiency assessment
There is no standardized EQA program for DM analyzers, making inter-laboratory comparisons difficult. The impact of DM analyzers on turnaround time and overall laboratory efficiency requires real-world studies to confirm whether they truly enhance the overall workflow without increasing risk.

Abbreviations: BF, body fluid; BM, bone marrow; DM, digital morphology; EQA, external quality assessment; PB, peripheral blood; PLT, platelet; RBC, red blood cell; WBC, white blood cell.
